# Semen collection, semen analysis and artificial insemination in the kākāpō (***Strigops habroptilus***) to support its conservation

**DOI:** 10.1371/journal.pone.0322276

**Published:** 2025-05-09

**Authors:** Dominik Fischer, Helena Schneider, Daryl Eason, Andreas Bublat, Deidre Vercoe, Fiona Robertson, Kākāpō Recovery Team, Bruce C. Robertson, Andrew Digby, Michael Lierz

**Affiliations:** 1 Clinic for Birds, Reptiles, Amphibians and Fish, Faculty of Veterinary Medicine, Justus Liebig University Giessen, Giessen, Germany; 2 Department of Conservation, Kākāpō Recovery, Invercargill, New Zealand; 3 Department of Zoology, University of Otago, Dunedin, New Zealand; Zagazig University, EGYPT

## Abstract

The critically endangered kākāpō (*Strigops habroptilus*) has suffered population declines due to habitat loss, hunting, and predation. Conservation efforts, including translocation to predator-free islands, have helped increase numbers of this flightless parrot from 51 individuals in 1995–142 in 2019. However, low fertility and high embryo mortality, likely due to genetic bottlenecks continue to hinder population growth. This is further aggravated by the kākāpō’s lek mating system, which allows only a minority of males to father a disproportionate number of offspring, resulting in 21% of non-reproductive males. The study aimed to enhance assisted reproduction techniques to assess male fertility, increase egg fertility, and ensure genetic diversity. Artificial insemination (AI) was used to mimic a second copulation, as females mating with multiple males show higher fertility rates. During the 2019 breeding season, semen collection was successful in 20 males and in 93.5% of 46 attempts using abdominal massage method and electric stimulation technique. Semen volume, colour, consistency, contamination, pH and the motility, concentration, viability and morphology of spermatozoa were analysed. Ejaculate volume ranged between 0.1 and 210 µl and the mean pH was 7.5 ± 0.4 ($\overset{―}{x}$ ± SD). Average sperm viability was 87.4 ± 10.0% with a total motility of 60.9 ± 22.0% and a progressive motility of 28.3 ± 19.8%. AI was performed 15 times in 12 females, improving second clutch fertility (70% vs. 29.4% without AI). Egg fertility in the second clutch without AI was 29.41% (5/17) compared to 70% (14/20) after AI. Paternity testing confirmed AI offspring (four chicks of three females), including from two previously non-reproductive males, enriching the gene pool with rare alleles (e.g., genes from Fiordland founding population). This study demonstrates the value of assisted reproduction in conserving endangered avian species by improving reproductive success and preserving genetic diversity.

## Introduction

The kākāpō (*Strigops habroptilus*) is a large flightless, nocturnal parrot endemic to New Zealand. They weigh up to 4 kg and are usually solitary with an individual range of aprroximately 0.15 km^2^ with a high variation [1]. They inhabit forests and scrublands where they feed on fruits, seeds, buds and roots of various plants [2].

### Population/threat status of the kākāpō

The kākāpō is regarded as one of the world’s rarest birds, regarded as critically endangered by the IUCN [3], with only 142 individuals being alive in 2019 [4]. Kākāpō are vulnerable to introduced mammalian predators because their camouflaged plumage and nocturnal habits are an ineffective protection against predators, which hunt by smell. Such mammalian predators were introduced by humans as neozoan invasive species in New Zealand [5–7]. During the 1970s to 1990s all the surviving kākāpō were transferred from the two last natural populations in Fiordland and on Rakiura/Stewart Island to three predator-free islands [6,8].

### Kākāpō reproduction biology

Breeding is dependent on the episodic mast fruiting of trees such as the rimu (*Dacrydium cupressinum*), which typically occurs only once every 2–5 years [9,10].

Unlike other parrots, kākāpō have a polygynous lek mating system. During breeding years most (≥ 80%) adult males (≥ 5 years) continuously display, using booming (noise created by using their air sacs) and chinging (vocalizing) throughout the nights from mid December to late March at their exclusive track and bowl site to attract females [11]. Previous records of the Kākāpō Recovery Team on mating events detected an average copulation time of 39 minutes (n = 17; SD 10; range 16–54 minutes). Female breeding behaviour begins later than males and is characterized through one to four copulations with one to three males prior to egg laying [11]. The average clutch size is 2.45 (range 1–4) eggs and the egg laying interval is generally 2.5 to 3.5 days [10]. The females are solely responsible for incubation and chick rearing and leave their nests unattended for multiple hours at night when they search for food. Thus, eggs and chicks are vulnerable to hypothermia and predators [10].

Despite removing the threat of predators, the population’s growth has been hampered by low fertility rate of eggs and a high incidence of early embryo deaths [7,12,13]. Average fertility has been reported to be 67.4% and average hatchability 43% [11].

A likely reason may be a low genetic variability [14], which is also known in other highly endangered parrot species such as the Spix’ Macaw (*Cyanopsitta spixii*) [15].

In the kākāpō it is known that hand-rearing and copulation behaviour have an effect on fertilization success. Females copulating with multiple males had the highest chance to produce a fertile clutch with 84% if the father was wild-reared and 66% with a hand-reared father, followed by females repeatedly copulating with one male (72% with wild-reared father and 64% with hand-reared father), whereas females copulating only once had the lowest reproduction success (50% with wild-reared father and 39% with hand-reared father) [16]. That multiple copulation leads to a higher percentage of fertile eggs is most likely due to sperm competition of the semen within the female reproductive tract [15]. Presently the male selection process by the females is unknown [17], but by preferring certain kākāpō some kākāpō males have never reproduced and thus have not transferred their genes to any offspring [18]. These males included founder males carrying the only remaining genetics from Fiordland, which are potentially vital to the population. Assisted reproduction (AR) techniques like semen collection and artificial insemination (AI) have been described as a useful tool in captive breeding of parrots [19–22] and were previously used in the kākāpō [23–25]. As with other parrot species [26,27], semen collection in kākāpō has been achieved through a modified abdominal massage technique [23], but a success rate of semen collection in the kākāpō has not been reported and most semen parameters are presently unknown.

AI using fresh semen has been attempted at least 24 times in 19 female kākāpō (2009, 2011, 2014 and 2016) and resulted in five fertilized eggs laid by two females in 2009, but not in fertile eggs in the following breeding years [23]. In various parrot species, semen collection and semen evaluation have helped to investigate infertility of males as a potential cause of egg infertility [28]. Also, in the kākāpō, it would be possible to identify infertile males and to remove them from the island to offer fertile males a better mating chance. Additionally, artificial insemination using semen of males the female did not copulate with, would mimic the situation of a female copulating with different males and thus might increase the clutch fertility rate, potentially due to the positive effect of sperm competition [29]. Last but not least, assisted reproduction can assist in maintaining the genetic diversity in the population by using semen of males which have not fathered offspring before or which carry rare genetics. AI is therefore also a tool for genetic management as demonstrated in other avian species [21,30]. Recently, a novel semen collection technique to collect semen of large parrots by electric stimulation has been developed [22], which has not been used in the kākāpō to date. This technique could also help to increase the success of semen collection, especially in those males providing no or little semen by massage method.

The development of short-term storage protocols and the investigation of semen diluents [30,31] are important to maintain sperm viable during transport on the island and thus to facilitate successful AI. Moreover, by adding a suitable semen extender and thus by increasing the sample volume, samples of genetically high-priority males may be split and used for several females in order to spread their genetic information and to increase the probability of offspring [19,22]. In addition, the results of semen analysis are the base for the development of a cryopreservation protocol, which hopefully will facilitate a prolonged preservation of the kākāpō’s genetic information in future.

The overall aim of the present study was the promotion of assisted reproduction techniques in the kākāpō, especially semen analysis and artificial insemination as part of the kākāpō recovery project on Whenua Hou/Codfish Island. In particular, the focus was on improvement of the egg fertilization rate and improving the reproductive success of males which had no offspring before.

## Materials and methods

### Animals and project site

The project site was Whenua Hou/Codfish Island, New Zealand where 29 adult female and 27 adult male kākāpō lived free-ranging during the study period (Jan – Mar 2019). The abundance of rimu fruit meant that the year 2019 was expected to be a breeding year in which most, if not all adult animals were expected to breed. The breeding season started in late December 2018 and the average mating period was supposed to last nine weeks, with the peak mating in the first half of February. The males were categorized into three different categories of semen donors regarding to their genetic origin, namely category A (highest priority; Fiordland origin), category B (lower priority; Stewart Island founders with no progeny) and category C (lowest priority; Stewart Island founders and their offspring with progeny). Additionally, for each female matching males were assigned based on their genetic relatedness using a unpublished Genetic Relatedness Matrix (GRM) (Robertson unpublished data) [32,33]. Only semen of males with a low relatedness score to the target female was used for artificial insemination in order to prevent inbreeding [18].

All kākāpō were free ranging and equipped with a radio transmitter (Lotek, Havelock North, New Zealand) which allowed them to be located and which contained sensors to detect and record copulations. These logged the date and time of copulation, which individuals were involved, and a quality score [7]. This copulation information was transmitted to the internet via data loggers (“snarks”) located across the island, providing a record of copulations each morning.

For semen collection or artificial inseminations birds were tracked by their radio transmitters and caught by sight and put in a washable cotton bag in order to determine their body weight using a digital luggage scale prior to further handling. A brief health check of each bird was performed prior to further handling, paying special attention to the general condition and the absence of injuries [34].

### Semen collection

**Fig 1:**
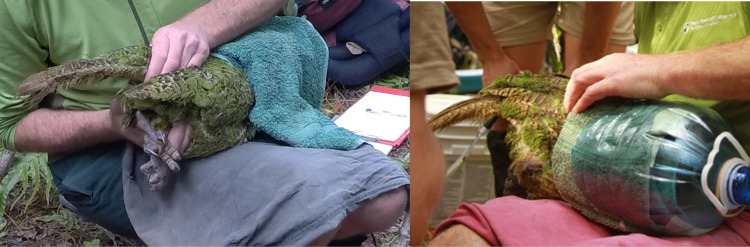
Semen collection in kākāpō using abdominal massage technique from two different points of view. A male kākāpō is manually restraint, carefully wrapped in a towel and with the head and chest inserted in a bottom removed water bottle.

**Fig 2:**
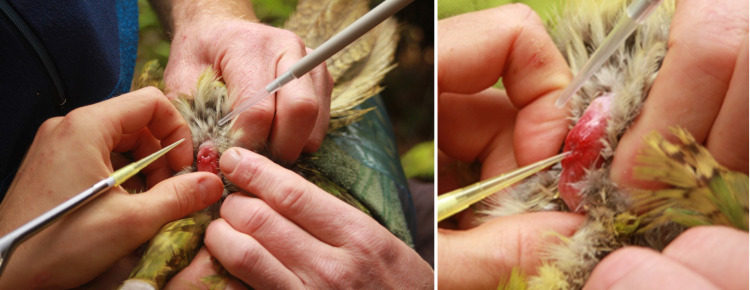
Semen collection from the cloaca of a kākāpō using pipettes and plastic pipette tips. Using thumb and index finger the cloaca is gently pressed to carefully squeeze the semen out of the cloaca. Assistants help to hold feathers away in order to prevent that semen is soaked up by the feathers.

During the 2019 breeding season, semen collection was attempted 46 times from 20 different males. Semen was collected from male kākāpō either via the massage method [35], or additionally by the use of a multipolar probe (patent-pending; JLU Giessen, Germany) [22]. To perform the massage, the males were held in ventral recumbency on the lap of the semen collector. To avoid defensive movements of the bird, the head and the wings were covered with a towel and the first half of the bird was inserted into a plastic bottle for protection (Fig 1). The feet were restrained with one hand as an extension of the back and if necessary, their cloaca was cleaned using tissues to reduce the chances of contaminating the sample with feces. The thumb and the fingers of the other hand were positioned on both sides of the cloaca and gentle opposed rhythmic movements of the thumb and fingers in a “squeezing” fashion on the abdomen of the bird induced ejaculation in some cases. If an active ejaculation could not be evoked, semen was gently pressed out of the seminal ampules of the deferent duct by massaging the abdomen (Fig 2) [35].

**Fig 3:**
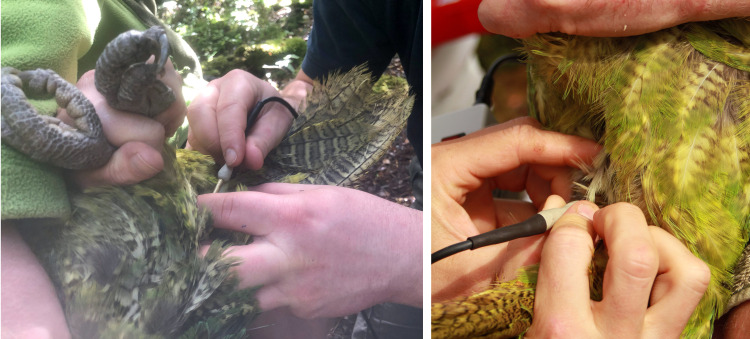
Semen collection in kākāpōs using electric stimulation. A bodysize-adapted multipolar probe was inserted into the urodeum of the cloaca in order to stimulate the ampullae ductus deferentes and to collect semen using a slight electrical current.

If no semen could still be collected, electric stimulation was attempted. Therefore, a bodysize-adapted probe was inserted into urodeum of the cloaca (Fig 3) in order to stimulate the *ampullae ductus deferentes* using a slight electrical current in three short impulses (duration of each impulse 1 s). The voltage was increased gradually from 0.09 V to a maximum of 2.7 V until contractions of the cloaca and the muscles of the tail were observed; each impulse was followed by a 2–3 s break.

Following both methods, semen was collected by applying a gentle squeezing-pressure to the cloaca with the thumb and index finger, causing a slight protrusion of the cloaca. Semen was then collected into 1000 µl pipet tips (Pipet tip MAKRO 0.1–2 ml, Carl Roth GmbH + Co. KG, Karlsruhe, Germany) and then transferred into an Eppendorf® Safe-lock microcentrifuge tube (Eppendorf AG, Hamburg, Germany). After semen collection, the restrain was dissolved, the male was rewarded with a nut and released.

### Semen analysis and evaluation of different semen diluents

**Fig 4:**
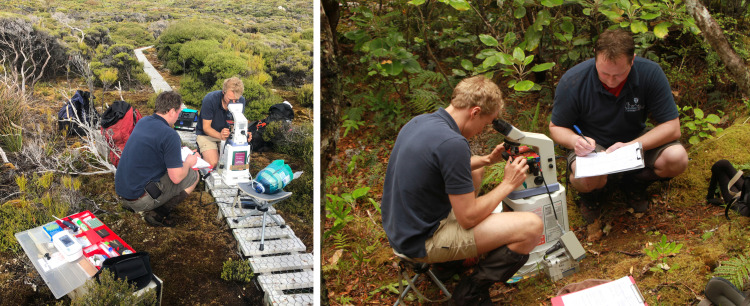
Semen analysis was performed immediately in the field using mobile equipment. This included a microscope with 40 X - 400 X magnification and phase contrast and a heating pad attached to a 12V mobile motorcycle battery. The individuals depicted in this manuscript have given written informed consent (as outlined in PLOS consent form) to publish this picture and these case details.

Semen evaluation was performed using mobile equipment in the field and included the assessment of volume, color, and density of semen, as well as motility and viability of the spermatozoa. Sperm density and motility were estimated immediately after collection. Therefore, 1 µl of semen was drawn into a graded microcapillary (Wiretrol I, Drummon Scientific Company, Broomall, PA, USA) and examined using a Nikon YS100 microscope with 40 X - 400 X magnification and phase contrast (Nikon Corporation, Tokyo, Japan), equipped with a heating pad (RS Pro Silicone Heater Pad 245–534, RS Components Ltd., Corby, Northants, UK) attached to a 12V mobile motorcycle battery. Semen evaluation, except for sperm morphology and semen storage study, was performed immediately in the field (Fig 4)

Firstly, an estimation of the amount of spermatozoa was performed by estimating the density of spermatozoa inside the microcapillary and by assigning it to a category from 0 (very few spermatozoa, less dense sample) to 5 (massive/tightly packed, very dense spermatozoa sample) as described before [31].

Sperm velocity was assessed by judging the velocity of spermatozoa moving towards the liquid air barrier in the microcapillary tube and classified from 0 (no movement) to 4 (fast movement) at 37 °C as described in a previous field study [30].

A part of the semen sample was immediately diluted (1:200) with pre-warmed (37° C) physiological sodium chloride solution (Isotone Kochsalz-Lösung 0.9% Braun, B. Braun, Melsungen AG, Melsungen, Germany) to estimate sperm motility. From this, 8 µl of the diluted semen was placed on a slide covered with a cover slip. Thereafter, in a defined area of each five visual fields, spermatozoa were counted as previously described [36] and classified as non-motile and motile spermatozoa (MOT), whereby motile sperm were differentiated by forward progression (PMOT), local motility and circular motility.

The pH was measured from a small drop of undiluted semen (≤ 0.5 µl) by using indicator test-strips (Spezialindikatorpapier, pH 6.4–8.0, Macherey-Nagel GmbH & Co. KG, Dueren, Germany).

Sperm concentration was assessed in a Neubauer improved cell counting chamber following dilution of 2–5 µl semen with distilled water. Total sperm count (hereafter, TSC) was calculated by multiplication of the sperm concentration and ejaculate volume [37]. The dilution factor depended on the initial estimation of sperm density inside the capillary. For less-dense samples (categories 1 and 2), a dilution up to 200-fold was used, for more dense samples (categories 3 and 4) a higher dilution factor, up to 600 was applied.

For viability analysis, 2 µl of undiluted semen was mixed with the same amount of eosin B-2% on a glass slide, incubated for 15 s and smeared according to standard procedures [38]. In the dried smear, 200 spermatozoa were evaluated by differentiation of dead (colored) from live spermatozoa (unstained or white) to calculate a live/dead-ratio.

The smear was later preserved using a mounting medium for cover slips (Entellan™ new, Merck KGaA, Darmstadt, Germany) and a morphologic evaluation of 200 spermatozoa in the smear was performed at 1000 x magnification using oil immersion and a Nikon Eclipse E200 microscope (Nikon Corporation, Tokyo, Japan) or a Leica DM750 microscope (Leica Mikrosysteme Vertrieb GmbH, Wetzlar, Germany), respectively. Spermatozoa were classified into morphologically normal and abnormal spermatozoa, based on a classification scheme in ganders [39], which has been used in several taxonomically diverse avian species [20,21,31,37,40]. Abnormal spermatozoa had one or more of the following characteristics: head, acrosome, midpiece, tail malformations or a combination of those.

Residual semen samples were stored in a portable fridge (Dison Portable Cooler Box BC 170A, Zhengzhou Dison Electric Co Ltd, China) at + 4 °C until further use for artificial insemination on the same day or taken to the base station if no AI was performed the same day. Semen of high-priority males was then added to a semen diluent in 1:2 dilution and stored in the fridge at + 4 °C if a matching female was due to be caught the next day. Semen samples of low priority males with no matching female available were diluted 1:4 with six different semen extenders in comparison and sperm motility and viability was re-evaluated after 24, 48, 72 and/or 96 h, respectively [31]. The following semen extenders were used: 1) NaCl 0.9% (Braun, Germany), 2) Glutac-2 (AMP-Lab GmbH, Muenster, Germany) and four modified semen extenders prepared according to semen extenders used in cockatiels (modified Blanco`s semen extender, 1% Glucose-Ringer`s® solution, modified BPSE [= Beltsville poultry semen extender] and modified Lake semen extender) [31]. Due to the field conditions – mainly regarding the limited planning capability of capture operations as well as multi-functional staff deployment – and limited semen volume, not every sample were evaluated with each of the six diluents.

### Artificial insemination, clutching and fertility rates

If a female copulated naturally with a male she was selected as a candidate for artificial insemination. As it was estimated that egg-laying was due 4–10 days after the first copulation [11,41], insemination was tried one or two times after a copulation was detected. One primary male and up to two alternative males were chosen for each female as semen donors, according to their genetic relationship and frequency of their alleles within the population. Additionally, those males must be different to the male she naturally copulated with to allow paternity testing of potential offspring and thus to evaluate the successful way of insemination (natural vs. artificial).

**Fig 5:**
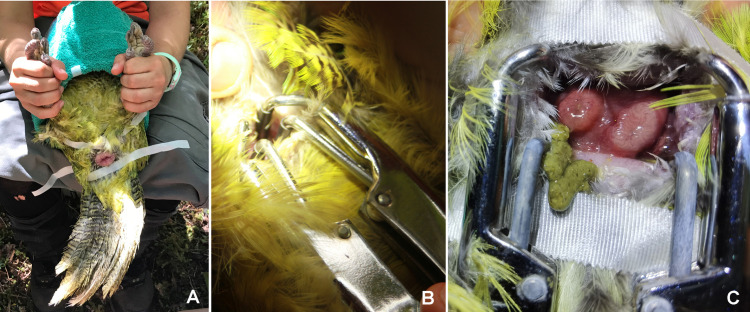
Artificial insemination in a kākāpō. A) Birds were restrained in dorsal recumbency with their head and chest elevated and their feet gently pulled towards their head. Feathers are taped out of the way using a weakly adhesive tape. B) Veterinary elastrator pliers were used for insertion into the cloaca, spreading the cloaca for visualization of the oviductal opening to enable semen deposition. C) Inside the spread cloaca the oviductal opening (left body side) must be recognized and differentiated from the intestinal opening (left body side), being slightly darker and may show feces in the lumen or directly in front as on this picture.

Female kākāpō chosen for artificial insemination were first restrained in ventral recumbency with their head and chest slightly lowered and their feet slightly pulled to both sides. Meanwhile, their head and wings were gently constrained using a towel to prevent wing flapping or other movements. During the second half of the study, the females were restrained in dorsal recumbency with their head and chest elevated and their feet gently pulled towards their head (Fig 5A). The cloaca was cleaned with moist tissues if faeces or uric contamination was visible. The examiner wore a strong headlamp for good visibility. A medium-sized speculum (65 mm; Henry Schein Services GmbH, Langen, Germany) or veterinary elastrator pliers (Demotec, Elastrator Instruments 865022, stainless steel, 22 cm, Eickemeyer, Tuttlingen, Germany) were used for insertion into the cloaca, spreading the cloaca for visualization of the oviductal opening to enable semen deposition (Fig 5B and 5C). For insertion into the vaginal opening, different-sized peripheral venous catheters (Vasofix® Braunüle® G16 – G18, 45 mm; B. Braun Melsungen AG, Melsungen, Germany) were used. Intravaginal injection of semen out of catheter was achieved either with help of air within a 1 ml syringe (Injekt®-F, B. Braun Melsungen AG, Melsungen, Germany) or a micropipette (Discovery Pro 10–100 µl, HTL Lab Solutions, Warszawa, Poland), respectively. After the procedure, the females were briefly restrained in the described position while the tail was gently articulated to simulate natural tail movements after copulation before they got rewarded and were released.

Reduced activity of female over time was interpreted as sign for nesting, and obtained from transmitter data. The females were tracked at their nests via telemetry and the nests were closely monitored regarding oviposition. Every egg of the clutch was removed from the nest and replaced by an artificial egg. Removed eggs were artificially incubated (Brinsea, Maxi Advance, Florida, USA) and evaluated for embryonic development by candling and weighing in order to monitor the weight loss during the incubation process. After the clutch was completed, in 19 of the 28 first clutches all eggs were removed to induce laying of a second clutch with the aim of increasing the number of eggs.

This study was done in accordance with national animal welfare and species conservation regulations, as part of the Kākāpō Recovery Program under the New Zealand Wildlife Act. Field site access was permitted and controlled by the government agency New Zealand Department of Conservation (DOC).

### Paternity testing

Paternity tests were performed on every chick or embryo using genotyping-by-sequencing (AgResearch Ltd) [33], for use in future genetic management of the population. Following hatch of each chick in an incubator, blood vessel tissue was taken from the soft membranes at the inner aspect of the eggshell. For the minority of chicks hatching in nests, a blood sample was collected after 1 month of age, or tissue from the dead embryo was taken, respectively, and submitted for genetic examination.

### Statistical analysis

Descriptive statistics of semen parameters including calculation of arithmetic mean, standard deviation, median, 25% and 75% quartile and range were performed using the program Excel for Windows (MS Office 365 Package, Microsoft Corporation, Redmond, WA, USA) and the program SAS 9.4 [42]. To prevent pseudoreplication due to repeated measures of the same individual, only one randomly selected sample per male was included in the descriptive analysis of semen parameters.

In a second assessment, semen sample collection via abdominal massage and electric stimulation was compared, followed by a comparison of various semen extenders and the maintenance of sperm quality during storage. These analyses were performed using mixed-effects model (Generalized Linear Mixed Model) in SAS 9.4 (SAS® Institute Inc., 2013. Base SAS® 9.4 Procedures Guide: Statistical Procedures, 2nd ed. Statistical Analysis System Institute Inc., Cary, NC, USA). Normality was checked by Shapiro-Wilk test, Kolmogorov-Smirnov test, Cramer-von Mises test and Anderson-Darlin test. To check for significant differences, in normally distributed datasets a paired t- test was used and in not normally distributed datasets a Wilcoxon signed-rank test or a Mann-Whitney U-test, respectively. To investigate if the contamination of semen, the semen diluent used or the duration of semen collection had any influence on semen parameters, a pairwise two-sided multiple comparison analysis was used. Additionally, Fisher’s hypergeometric test was used to assess relationship between fertility and insemination method (natural vs. artificial).

The following questions were addressed:

1.) Are there statistically significant differences in semen parameters in male kākāpō between the first semen collection and the following semen collections and between the methods used for semen collection (massage vs. electric stimulation)?
2.) Are there statistically significant differences in semen parameters (sperm viability, sperm motility) between the different semen extenders, assessed immediately after dilution and during storage of diluted semen samples?
3.) Is there a relation or a statistically significant difference in clutch fertility (minimum 1 egg is fertile) or in egg fertility, respectively, between females with AI and without AI? (Chi-share test, Fisher’s hypergeometric test, Wilcoxon test).

A level equal to or below α = 0.05 was regarded as statistically significant.

## Results

### Animals and project site

Between January 24, 2019 and February 24, 2019, 32 (*N *= 20 males; *N *= 12 females) adult kākāpō were captured for assisted reproduction techniques on Whenua Hou. According to their genetic priority, eleven males were caught multiple times for semen collection, while three females were caught twice for artificial insemination. Five males were captured twice, one male was captured three times, three males were caught four times, one male was caught five times and another “A” male was caught seven times. All birds were in a good body condition with no, or negligible injuries. The age of the male birds averaged 22.0 ± 11.7 years ($\overset{―}{x}$ ± SD) with a range between 7 and at least 39 years and their body weight ranged between 1.9 and 3.1 kg ($\overset{―}{x}$ ± SD = 2.7 ± 0.3; Table 1). Mean age of the females was 25.6 ± 10.9 years ($\overset{―}{x}$ ± SD) with a range between 8 and at least > 38 years and an average body weight of 1.6 ± 0.1 kg ($\overset{―}{x}$ ± SD; range 1.4–1.9 kg). Total handling times (from catch to release) of the male birds ranged between 11 minutes and 1 hour ($\overset{―}{x}$ ± SD = 25.0 ± 14.4 min; Table 1), whereas total handling of the females ranged between 6–34 minutes ($\overset{―}{x}$ ± SD = 18.4 ± 8.2 min).

**Table 1:** Mean body weight, age, handling times and semen parameters of male Kākāpō (*Strigops habroptilus*) (n = 20).

	BW [kg]	Age [y]	THT [min]	Time SC [min]	Vol [µl]	Sperm dens.	pH	Mot. est.	MOT [%]	PMOT [%]	Viab. [%]	Sperm. conc. [n/µl]	TSC [n]
**mean** **± SD**	2.7 ± 0.3	22.0 ± 11.7	25.0 ± 14.4	10.8 ± 5.6	30.7 ± 37.6	2.5 ± 1.3	7.5 ± 0.4	2.1 ± 1.5	60.9 ± 22.0	28.3 ± 19.8	87.4 ± 10.0	3.28 ± 3.60 x 10^6^	1.03 ± 1.44 x 10^8^
**range** **(min -** **max)**	1.9-3.1	7 – 39	11.0 – 95.0	3.0 – 30	0.0 - 210	0 – 4	6.4 – 8.0	0 – 4	0 – 90.0	0 – 72.0	52.0 – 99.0	0 – 1.20 x 10^7^	0 – 6.68 x 10^8^
**median**	2.7	20	21.0	10.0	16.5	2.0	7.6	2.0	62.0	22.0	89.0	8.93 x 10^5^	3.5 x 10^7^
**Q1**	2.5	9	16.5	7.0	5.8	1.6	7.4	1.0	50.5	14.3	84.4	2.39 x 10^5^	3.95 x 10^6^
**Q3**	2.9	33.5	28.0	12.8	42.3	4.0	7.8	4.0	79.5	37.0	95.0	6.25 x 10^6^	1.61 x 10^8^
**N**	37	19	42	42	46	46	44	46	31	31	35	40	40

Abbreviations: mean, arithmetic mean; SD, standard deviation; min, minimum value; max, maximum value; Q1, 1st quartile; Q3, 3rd quartile; BW, body weight; THT, Total handling time; Time SC, duration of semen collection; Sperm dens., estimated sperm density; Mot. est., estimated sperm motility; MOT, total sperm motility; PMOT, progressive sperm motility; Viab., viability; Sperm. Conc., sperm concentration; TSC, total sperm count; Sperm dens, estimated sperm density; N, number of investigations.

### Semen collection

Semen collection was attempted 46 times in 20 different males and was successful in 43 attempts (93.5%), regardless of collection method (i.e., massage [n = 46] or electric stimulation [n = 9]). Massage was tried first in all attempts and resulted in semen collection in 89.1% (41/46) of all cases. In most cases, male kākāpō responded to the rhythmic movements of the fingers during the massage method with leg movement and cloacal contraction. Nine times in eight males, electric stimulation was tried additional to massage and was successful in 88.9% (8/9) attempts. However, only in three out those nine attempts did initial massage not result in semen extraction, whereas after electric stimulation semen collection was achieved. Slight electric stimulation through the electric probe led to cloacal contractions and agglomeration of the toes. Neither procedure provoked defensive behavior of the birds. On average, semen collection lasted 10.8 ± 5.6 minutes ($\overset{―}{x}$ ± SD; Table 1).

### Semen analysis and evaluation of different semen diluents

Mean sperm density inside the capillary was 2.5 ± 1.3 ($\overset{―}{x}$ ± SD), while estimated sperm motility in the microcapillary tube was 2.1 ± 1.5 ($\overset{―}{x}$ ± SD) (Table 1). Fecal, uric and hemorrhagic contamination occurred in 39.5% of all semen samples (4 [= 23.5%] by uric 3 [= 17.6%] by fecal, 5 [= 29.4%] by erythrocytes and 5 [= 29.4%] were contaminated by two or more factors). Individual semen volume ranged from 0.1 to 210 µl, with an average of 30.7 ± 37.6 µl ($\overset{―}{x}$ ± SD). Semen pH ranged from 6.4–8.0 with an average of 7.5 ± 0.4 ($\overset{―}{x}$ ± SD; Table 1). The median sperm concentration was 8.93 x 10^5^ spermatozoa/µl (Q1 = 2.39 x 10^5^ spermatozoa/µl, Q3 = 6.25 x 10^6^ spermatozoa/µl; Table 1). Comparing both methods for semen collection, differences were obvious for sperm concentration (massage: $\overset{―}{x}$ ± SD = 3.52 x 10^6^ ± 3.58 x 10^6^ spermatozoa/µl; electric stimulation: $\overset{―}{x}$ ± SD = 5.85 x 10^4^ ± 4.40 x 10^4^ spermatozoa/µl; Table 2), but this difference was not statistically significant using paired t- test (p = 0.8125).

**Table 2:** Comparative semen evaluation of male Kākāpō (*Strigops habroptilus*) collected through two different methods of semen collection.

	Volume [µl]		Sperm dens. [n]		Velocity [n]		Occurrence of contamination [%]		pH		MOT. [%]		PMOT [%]		Via. [%]		Sperm concentration [n x 10^5/µl]		TSC [n x 10 ^8]	
	M	E	M	E	M	E	M	E	M	E	M	E	M	E	M	E	M	E	M	E
**mean ± SD**	24.9 ± 35.6	29.8 ± 32.7	2.5 ± 1.4	1.1 ± 0.8	2.1 ± 1.6	1.6 ± 0.7	34.1	62.5	7.5 ± 0.4	7.4 ± 0.3	64.0 ± 22.9	55.6 ± 13.6	31.1 ± 20.8	25.3 ± 19.3	88.5 ± 10.2	80.0 ± 15.3	35.2 ± 35.8	0.58 ± 0.44	1.08 ± 1.47	0.05 ± 0.08
**range** **(min – max)**	0 - 210	0 - 90	0 - 4	0 - 4	0 - 4	0 - 4	0: neg.; 1:pos.	0: neg.; 1:pos.	6.4–8.0	7.0–7.7	0 - 90	32 - 77	0 - 72	8 - 62	47.7 - 99	52 - 97	0–120.3	0.05–1.48	0–6.68	0.00006–0.254
**n**	46	9	46	9	46	9	43	8	39	8	28	7	28	7	31	7	34	7	34	7
**t test**	Wil		Ptt		Ptt		Ptt		Ptt		Ptt		Ptt		Ptt		Wil		Ptt	
***P***	0.1211		0.7275		> 0.05		0.1250		0.1253		0.8125		0.3830		0.4538		0.8125		0.2969	

Abbreviations: mean, arithmetic mean; SD, standard deviation; min, minimum value; max, maximum value; M, semen collection via massage; E, semen collection via electric stimulation; Sperm dens., estimated sperm density; Mot. Est., estimated sperm motility in the capillary; TSC, total sperm count; n, number of samples; P, p-value; Wil, Wilcoxon signed-rank test; Ptt, paired t test.

Semen samples solely collected through the massage method were contaminated in 34.1% of attempts (Table 2), of which three samples each (21.4%) were contaminated with erythrocytes or with feces. Contaminations with urates or combinations occurred in four samples (28.6%). Semen samples collected using electric stimulation were contaminated in 62.5% (5/8) of attempts (Table 2), with uric acid within 40.0% (2/5) samples. Other contaminations were detected in 20.0% (1/5) of samples. However, the degree of contamination as well as the pH value (p = 0.1253) were not significantly different between both methods of semen collection using a paired t- test. Using a pairwise two-sided multiple comparison analysis, urine contaminated semen samples were demonstrated to have significantly lower pH values (p = 0.0073) compared to semen samples free of contamination. No other statistically significant influence of semen contamination or the duration of semen collection on any semen parameter was demonstrated. Using a Wilcoxon signed-rank test no differences were detected for semen volume (p = 0.1211); and using paired t- tests, no significant differences were detected for sperm velocity (p > 0.05), sperm motility [MOT (p = 0.8120), PMOT (p = 0.3830)], sperm viability (p = 0.4538) and total sperm count (p = 0.2969) between the two methods of semen collection. The evaluation of individual semen parameters of repeatedly captured Kākāpō males is demonstrated in Table 3.

**Table 3:** Evaluation of individual semen parameters of repeatedly captured Kākāpō (*Strigops habroptilus*) males (n = 11), in ascending order of their genetic priority.

Male No.	Des. No.	Age [y]	Volume [µl]	Sperm dens.	Mot. Est.	pH	MOT [%]	PMOT [%]	Viab. [%]	Sperm conc. [n x 10^5/µl]	TSC [n x 10^5]
2	KP 12	> 38	10	4	2	8	60	38	80	42.75	427.5
	KP 30		12	2	1	7.2	n.i.	n.i.	87	5.1	61.2
15	KP 19	10	2	2	3	7.8	n.i.	n.i.	96	n.i.	n.i.
	KP 37		43	2	2	7	77	62	98	0.625	26.88
16	KP 20	> 39	20	2	1	7.6	31	16	92	0.70	14
	KP 40		1	0.5	2	7.6	50	8	52	0.06	0.06
17	KP 21	28	15	3.5	4	7.8	63	47	97	33.5	502.5
	KP 34		75	2	2	7.7	62	3	89	7.26	544.5
19	KP 28	> 30	85	2	1	6.5	n.i.	n.i.	69	n.i.	n.i.
	KP 38		210	0	0	6.4	n.i.	n.i.	n.i.	0.025	5.25
9	KP 10	21	24	4	4	7.8	81	36	79	64.75	1,554
	KP 26		8	0.5	0.5	n.i.	n.i.	n.i.	n.i.	n.i.	n.i.
	KP 44		12	2	2	7.6	32	18	84.5	1.48	17.7
1	KP 1	> 31	60	4	2	6.8	62	15	87	52.4	3,144
	KP 8		60	4	4	7.8	79	35	92	111.3	6,678
	KP 32		86	2	1.5	6.9	n.i.	n.i.	95	9.3	799.8
	KP 35		9	0.5	0	7.8	48	6	n.i.	n.i.	n.i.
4	KP 3	> 37	10	1	1	6.4	3	0	n.i.	n.i.	n.i.
	KP 24		15	3	1	7.4	49	7	78	3.95	59.25
	KP 42		1	n.i.	n.i.	7.0	n.i.	n.i.	n.i.	n.i.	n.i.
	KP 45		4	0.5	0	7.2	n.i.	n.i.	n.i.	0.007	0.03
7	KP 7	> 30	18	4	4	8	77	21	89	84.9	1,528
	KP 13		13	4	4	7.8	72	55	89	61.8	802.8
	KP 23		12	4	3.5	7.8	n.i.	n.i.	n.i.	67.8	813.0
	KP 31		30	4	4	7.6	81	34	96	120.3	3,609
3	KP 2	> 37	30	4	4	7.8	90	70	99	60	1,800.00
	KP 11		28	4	4	8	84	33	92	73.75	2,065.00
	KP 25		0	n.i.	n.i.	7.4	n.i.	n.i.	n.i.	n.i.	n.i.
	KP 33		52	4	4	7.6	84	22	97	34.50	1,794.00
	KP 41		2	0.5	2	7.4	57	14	65	0.055	0.11
5	KP 4	21	35	4	4	7.9	86	22	90	64.60	2,261.00
	KP 9		0	n.i.	n.i.	n.i.	n.i.	n.i.	n.i.	n.i.	n.i.
	KP 22		40	4	4	7.8	84	72	98	97.48	3,899.00
	KP 29		45	2	1	7.8	n.i.	n.i.	87	3.73	167.63
	KP 36		67	2	2	7.8	51	13	97	3.80	254.60
	KP 43		70	2	2	7.8	46	15	79	0.625	43.75
	KP 46		0.1	0.5	0	7.4	n.i.	n.i.	n.i.	0.02	0.001

Abbreviations: No, number; Des., desemination; Sperm dens, estimated sperm density; Mot. est., estimated sperm motility; MOT, total sperm motility, PMOT, progressive sperm motility; Viab, viability; Sperm conc., sperm concentration; TSC, total sperm count; n.i., not investigated.

Morphological evaluation of 33 semen samples (i.e., 200 sperm cells) was performed. The rest of the samples did either not contain a sufficient number of spermatozoa for a sound evaluation, or no stained sample was produced due to a limited sample volume and use for AI and were therefore excluded from the analysis. Morphological abnormalities mainly affected the tail ($\overset{―}{x}$ ± SD = 27.1 ± 13.2%; Table 4) and the head regions ($\overset{―}{x}$ ± SD = 11.0 ± 7.7%), followed by abnormalities in the mid-piece section ($\overset{―}{x}$ ± SD = 8.9 ± 7.0%) and the acrosome ($\overset{―}{x}$ ± SD = 0.9 ± 1.0%). Multiple malformations within one spermatozoon occurred in 8.6 ± 5.8% ($\overset{―}{x}$ ± SD) of the morphological abnormal spermatozoa. A wide range (6.0–77.5%) of morphologically normal sperm cells (MNS) was detected in the evaluated semen samples (Table 4).

**Table 4:** Statistical evaluation of the number of morphological semen characteristics per slide of male Kākāpō (*Strigops habroptilus*). In each slide (N = 33) 200 spermatozoa were evaluated.

	Normal spermatozoa		Head abn. [n]		Acrosome abn. [n]		Midpiece abn. [n]		Tail abn. [n]		Multiple abn.	
	[n]	[%]	[n]	[%]	[n]	[%]	[n]	[%]	[n]	[%]	[n]	[%]
mean ± SD	86.2 ± 38.3	43.5 ± 18.3	21.7 ± 15.7	11.0 ± 7.7	1.7 ± 2.0	0.9 ± 1.0	17.1 ± 13.8	8.9 ± 7.0	53.9 ± 28.1	27.1 ± 13.1	16.6 ± 11.5	8.6 ± 5.8
range (min – max)	12 - 155	6 - 77.5	3 - 75	1.5- 37.5	0 - 9	0 - 4.5	2 - 65	1 - 32.5	12 - 115	10.5 - 56	2 - 45	1.0 - 22.5
median	90.0	44.6	17.0	9.4	1.0	0.5	13.0	6.5	49.0	13.0	49.0	6.5
Q1	57.0	32.0	8.0	5.4	0	0	9.0	4.3	32.0	9.0	32.0	4.5
Q3	113.0	56.5	27.0	13.5	3.0	1.5	23.0	11.5	74.0	17.0	74.0	13.1

Abbreviations: abn., abnormalities; mean, arithmetic mean; SD, standard deviation; min, minimum value; max, maximum value; Q1, first quartile; Q3, third quartile.

**Fig 6:**
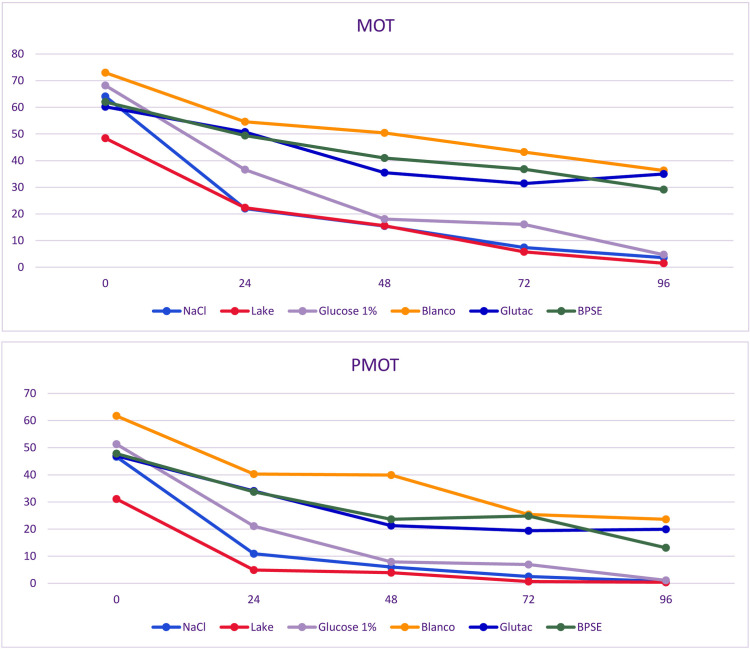
Assessment of sperm motility in diluted semen samples from kākāpō comparing fresh semen (Time point 0) to stored-semen after 24, 48, 72, and 96 hours storage time at + 4 °C in a refrigerator. As diluents isotonic saline (NaCl) 1), Glutac-2 (Glutac), modified Blanco`s semen extender (Blanc), 1% Glucose-Ringer`s® solution (Glucose 1%), modified Beltsville poultry semen extender (BPSE) and modified Lake semen extender (Lake) were used. Motility (MOT) depicts the percentage of all motile spermatozoa compared to the total number of spermatozoa, whereby the percentage of progressively forward moving Spermatozoa from all spermatozoa is depicted in the progressive forward motility graph (PMOT).

**Fig 7:**
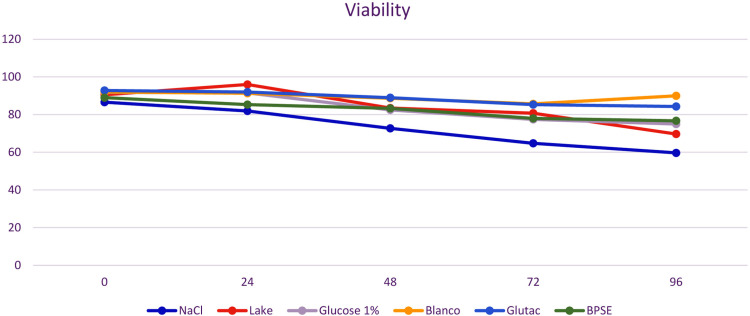
Assessment of sperm viability using Eosin B stain in diluted semen samples from kākāpō, comparing a fresh semen (Time point 0) to stored semen after 24, 48, 72, and 96 hours storage time at + 4 °C in a refrigerator. As diluents isotonic saline (NaCl) 1), Glutac-2 (Glutac), modified Blanco`s semen extender (Blanc), 1% Glucose-Ringer`s® solution (Glucose 1%), modified Beltsville poultry semen extender (BPSE) and modified Lake semen extender (Lake) were used. The percentage of viable spermatozoa is depicted compared to the total number of spermatozoa.

In total, 14 semen samples were sufficient to test short-term storage with different semen extenders. Mean volume of those residual samples was 68.6 ± 36.4 µl ($\overset{―}{x}$ ± SD), but only three semen samples were sufficient in volume and quality to be evaluated with each of the six diluents simultaneously. All other samples were limited in volume and were therefore only mixed with four or fewer semen extenders at the same time. In total, ten semen samples were added to physiological saline solution, eight samples to modified Blanco`s semen extender, seven samples to 1% Glucose-Ringer`s® solution, six samples to modified Beltsville Poultry Semen Extender, five to Glutac-2, and another four semen samples added to modified Lake semen extender respectively (Table 5, Figs 6 and 7). Using modified Blanco`s semen extender kākāpō semen quality could be maintained over a period of 96 hours guaranteeing highest sperm motility ($\overset{―}{x}$ ± SD = 36.35 ± 25.03% [MOT]; ($\overset{―}{x}$ ± SD = 23.37 ± 18.98% [PMOT]) and viability ($\overset{―}{x}$ ± SD = 84.25 ± 9.96%) values compared to other semen extenders (Table 5, Fig 6). Using Glutac-2, some semen parameters could also be maintained over 96 hours on acceptable levels (motility: $\overset{―}{x}$ ± SD = 26.58 ± 23.57% [MOT]; $\overset{―}{x}$ ± SD = 14.97 ± 19.72% [PMOT]; viability: $\overset{―}{x}$ ± SD = 75.50 ± 21.50%), similar to using BPSE (motility: $\overset{―}{x}$ ± SD = 26.58 ± 17.68% [MOT]; $\overset{―}{x}$ ± SD = 7.87 ± 11.18% [PMOT]; viability: $\overset{―}{x}$ ± SD = 76.20 ± 10.90%), but the use of physiological saline, 1% Glucose-Ringer`s® solution and Lake semen extender did not aid in preservation of semen quality.

**Table 5:** Reevaluation of MOT, PMOT and viability using different semen extenders in Kākāpō (*Strigops habroptilus*) semen samples under the aspect of storage time (in hours (h).

		mean ± SD		
	h	MOT [%]	PMOT [%]	Viability [%]
**0.9% - NaCl Solution** (n = 10)	0	63.95 ± 11.57	48.67 ± 12.94	86.50 ± 9.17
	24	17.69 ± 13.41	8.04 ± 8.89	80.69 ± 13.66
	48	10.63 ± 14.67	3.68 ± 7.23	71.56 ± 12.47
	72	5.12 ± 8.48	0.63 ± 1.77	64.06 ± 16.36
	96	0.41 ± 1.08	0.00 ± 0.00	50.00 ± 12.39
**Modified Blanco`s SE** (n = 8)	0	60.47 ± 16.41	41.09 ± 24.32	88.00 ± 9.16
	24	45.43 ± 16.03	28.15 ± 21.04	84.64 ± 10.30
	48	37.23 ± 17.53	19.93 ± 19.89	81.00 ± 12.86
	72	39.22 ± 18.54	20.04 ± 18.19	79.17 ± 13.11
	96	36.35 ± 25.03	23.37 ± 18.98	84.25 ± 9.96
**1% Glucose-Ringer`s ® Solution** (n = 7)	0	68.20 ± 10.60	51.32 ± 12.17	91.80 ± 8.66
	24	30.55 ± 22.49	17.58 ± 14.95	73.20 ± 37.12
	48	14.48 ± 16.78	6.35 ± 7.97	79.60 ± 11.83
	72	12.91 ± 15.82	5.52 ± 6.81	75.00 ± 9.98
	96	3.73 ± 7.47	0.90 ± 1.79	58.75 ± 29.66
**Modified BPSE** (n = 6)	0	50.74 ± 20.34	36.23 ± 21.38	89.00 ± 4.95
	24	39.87 ± 17.00	25.30 ± 15.39	89.50 ± 6.58
	48	33.25 ± 10.56	15.32 ± 11.30	80.40 ± 9.71
	72	34.12 ± 9.20	15.98 ± 13.65	75.3 ± 11.59
	96	26.55 ± 15.81	7.87 ± 10.00	76.20 ± 9.74
**Glutac-2 SE** (n = 5)	0	60.21 ± 15.09	47.30 ± 16.20	92.75 ± 4.76
	24	50.67 ± 11.91	33.99 ± 17.44	92.00 ± 6.36
	48	32.23 ± 17.99	17.77 ± 15.94	82.20 ± 14.99
	72	27.75 ± 17.15	15.12 ± 16.43	81.70 ± 12.87
	96	26.58 ± 20.41	14.97 ± 17.08	75.50 ± 18.62
**Modified Lake SE (n = 4)**	0	48.41 ± 8.88	31.08 ± 8.68	90.50 ± 8.65
	24	22.31 ± 8.20	4.93 ± 2.03	96.00 ± 2.45
	48	15.63 ± 6.72	3.87 ± 3.24	83.50 ± 10.11
	72	6.33 ± 4.42	1.32 ± 1.37	79.00 ± 13.15
	96	1.45 ± 2.05	0.39 ± 0.56	69.67 ± 24.57

Abbreviations: mean, arithmetic mean; SD, standard deviation; MOT, total sperm motility; PMOT, progressive sperm motility; Viab., viability; SE, semen extender; n, number of samples investigated.

### Artificial insemination, clutching & fertility rates

Twelve of 28 females were artificially inseminated (12 females once and 3 females twice) (Table 6) additional to natural copulation, while 16 females were not artificially inseminated. Five of the twelve females (41.7%; females no. 1–5) were artificially inseminated prior to the first egg of the first clutch (Table 7), and two females (females no. 2 and 3) were additionally inseminated a second time before the first clutch was completed (one of them again prior to the first egg (female no. 2) and the other one prior to the second egg (female no. 3)). Seven of the twelve females (58.3%; females no. 6–12) were artificially inseminated prior to the second clutch (Table 7), and one female (female no. 11) twice prior to oviposition of the second clutch.

**Table 6:** Mean body weight, age and handling times of female Kākāpō (*Strigops habroptilus*) (n = 12), as well as standard semen parameter of semen samples used for artificial insemination.

	Body Weight [kg]	Age [y]	Total Handling Time [min]	Dur. AI [min]	Insem. Volume [µl]	Sperm conc. [n x 10^6/µl]	Total Insem. Dose [n x 10^8/µl]	MOT [%]	PMOT [%]	Viab. [%]
mean ± SD	1.6 ± 0.1	24.0 ± 10.2	18.4 ± 8.2	3.5 ± 1.8	49.8 ± 25.8	5.90 ± 3.47	1.51 ± 1.51	64.4 ± 24.3	31.4 ± 19.3	90.8 ± 5.8
range (min–max)	1.4–1.9	8.0–38.0	6–34	1–7	15–112	0.07–12.03	0.007–6.68	6–86	2–72	79–98
median	1.6	27.0	17.5	3.0	50.0	6.32	1.07	77	33	92
Q1	1.5	15.5	12.5	2.0	30.0	3.45	0.58	60	19	89
Q3	1.6	31.0	24.0	5.0	65.0	8.21	1.91	81	38	96
N	11	12	14	15	15	15	15	14	14	14

Abbreviations: mean, arithmetic mean; SD, standard deviation; min, minimum value; max, maximum value; Q1, 1^st^ quartile; Q3, 3^rd^ quartile; Dur. AI, duration artificial insemination; Insem., insemination; Sperm. Conc., sperm concentration; MOT, total sperm motility; PMOT, progressive sperm motility; Viab., viability; N, number of investigations.

**Table 7:** Artificial inseminations (n = 15) performed in Kākāpō (*Strigops habroptilus*) females (n = 12), including semen quality parameter of semen samples and semen extenders used, as well as egg fertilization successes through AI (proven through paternity testing).

Female No.	Insem. No.	Age [y]	Total Handling Time [min]	Dur. AI [min]	Insem. Volume [µl]	SE	Sperm conc. [n x 10^6/µl]	Total Insem. Dose n x 10^8/µl]	Ø MOT [%]	Ø PMOT [%]	Ø Viab. [%]	No. Semen Samples/Insem. [n]	Fertilization Success AI [n]
First Clutch													
1	KPI 1	> 38	26.	4	30	/	6.00	1.80	15	65	82	1	0
2	KPI 2	38	34	5	30	/	6.46	1.94	86	22	90	1	0
	KPI 6		17	1	25	/	7.38	1.84	84	33	92	1	0
3	KPI 3	10	6	3	15	/	8.49	1.27	77	21	89	1	0
	KPI 7		14	2	50	G	2.46	1.23	66	47	85	2	0
4	KPI 4	27	24	2	60	/	11.13	6.68	79	35	92	1	0
5	KPI 5	30	16	2	20	/	6.48	1.30	81	36	79	1	0
Second Clutch													
6	KPI 8	17	10	5	35	G	1.46	5.10	50	32	91	2	0
7	**KPI 9**	**> 39**	**29**	**5**	**40**	**NaCl**	**9.75**	**1.95**	**84**	**72**	**98**	**1**	**2**
8	KPI 10	20	27	5	112	G + NaCl	2.47	2.76	6	2	89	2	0
9	**KPI 11**	**> 32**	**12**	**6**	**80**	**Blanco**	**12.03**	**3.61**	**81**	**34**	**96**	**1**	**2**
10	KPI 12	14	24	2	50	Blanco	3.45	0.86	84	22	97	1	0
11	**KPI 13**	**> 34**	**18**	**7**	**70**	**/**	**0.73**	**0.51**	**62**	**3**	**89**	**1**	**1**
	KPI 15		6	2	80	Blanco	0.40	0.12	51	14	97	1	0
12	KPI 14	8	20	2	50	Blanco	3.45	0.86	80	19	95	1	0

Abbreviations: No, number; Insem., insemination; Dur., duration; AI, artificial insemination; SE, semen extender; Sperm conc., sperm concentration; MOT, total sperm motility, PMOT, progressive sperm motility; Viab, viability; G, 1% Glucose-Ringer`s ® solution; NaCl, Sodium chloride solution 0,9%; Blanco, modified Blanco`s Semen Extender; AI samples leading to confirmed fertilization written in bold.

Veterinary elastrator pliers enabled a good view inside the cloaca of the female kākāpō. It was spread until the oviductal opening was visible on the left ventral aspect of the urodeum (Fig 5C). Using a peripheral venous G16-18 x 45 mm intravenous catheter, 0.5–1 cm insertion into the oviductal opening and deposition of the semen sample was feasible using air pressure by pressing down a micropipette or a 1 ml syringe, connected to the catheter. The handling times for AI procedure ranged between 1–7 minutes (Table 6). In 80% of the cases artificial insemination were performed intravaginally, while in three cases (20%) the vaginal openings were difficult to see and the insemination had to be performed intracloacally.

Eight artificial inseminations were performed using undiluted semen samples, while seven AIs were performed using specifically diluted semen samples (Table 7), either because semen samples of more than one male were pooled for AI or because semen samples had to be stored when the female could not be caught on the same day when the semen was collected. AI females received a median volume of 50 µl (Q1 = 30 µl, Q3 = 65 µl) containing 6.3 x 10^6^ spermatozoa/µl ($\tilde{x}$; Q1 = 3.5 x 10^6^ sperm/µl, Q3 = 8.2 x 10^6^ sperm/µl) (Table 6). The total insemination volume was 1.3 x 10^8^ spermatozoa/sample ($\tilde{x}$; Q1 = 8.6 x 10^7^ sperm/sample, Q3 = 1.9 x 10^8^ sperm/µl). Regarding semen quality, 90.8 ± 5.8% ($\overset{―}{x}$ ± SD) of the spermatozoa were viable with a mean MOT of 64.4 ± 24.3% ($\overset{―}{x}$ ± SD) and a mean PMOT of 31.4 ± 19.3% ($\overset{―}{x}$ ± SD) immediately prior to AI (Table 6).

All females started or continued their nesting behavior following AI and laid a minimum of two eggs per clutch. In total on Whenua Hou, 126 eggs were laid in 41 clutches by 28 females (Table 8), 89 eggs in 28 first clutches (N = 28 females) and 37 eggs in 13 second clutches (N = 13 females).

**Table 8:** Clutch and egg fertility rates in two groups (AI, n = 5; Control, n = 7) of female Kākāpō (*Strigops habroptilus*) differentiated between first and second clutch.

		Fertile Clutches [n]	Infertile Clutches [n]	Sum Clutches [n]	Fertile Clutches [%]	Total Clutch Fertility [%]	Fertile Eggs [n]	Infertile Eggs [n]	Sum Eggs [n]	Fertile Eggs [%]	Total Egg Fertility [%]
AI	1^st^ Clutch	2	3	12	40.00	66.67	7	8	35	46.67	60.00
	2^nd^ Clutch	6	1		85.71		14	6		70.00	
Control	1^st^ Clutch	13	10	29	56.52	58.62	36	38	91	48.65	45.05
	2^nd^ Clutch	3	3		50.00		5	12		29.41	

Abbreviations: AI, artificial insemination; n, number; differences are statistically not significant.

The total clutch fertility (a fertile clutch contains at least one fertile egg) was 58.62% (17/29) without AI, compared to 66.67% (8/12) clutch fertility following AI. In Fisher’s hypergeometric test no relation (p = 0.7342; odds ratio = 0.71) between fertility and either natural insemination or AI was detected. Differentiating the results between first and second clutch, the clutch fertility for the first clutch was 60.87% (14/23) without AI and 40.0% (2/5) with AI. In Fisher’s hypergeometric test no relation (p = 0.6239; odds ratio = 2.26) was detected between fertility and either natural insemination or AI for the first clutch. For the second clutch, clutch fertility was 50% (3/6) without AI and 85.71% (6/7) with AI (Table 8). In Fisher’s hypergeometric test no relation (p = 0.2657; odds ratio = 0.19) between fertility and either natural insemination or AI was detected for the second test.

From visual inspection (`candling’), total egg fertility was 45.05% (41/91) without AI and 60% (21/35) after AI. In Mann-Whitney U-test no significant difference (p = 0.3538) was detected between natural insemination and AI. Differentiating the results between first and second clutch, the egg fertility in the first clutch was 48.65% (36/74) without AI and 46.67% (7/15) after AI. In Mann-Whitney U-test no significant difference (p = 0.7952) was detected between both ways of insemination. In the second clutch fertility of eggs without AI was 29.41% (5/17) compared to 70% (14/20) after AI. (Table 8). In Mann-Whitney U-test no significant difference (p = 0.0906) was detected between natural insemination and AI, but the low p-value points towards an effect. Results of paternity test confirmed that four of eight eggs laid by three females (females no. 7, 9 and 11) in their second clutch were fertilized by AI. In one female (female no. 9) even two of three eggs were fertilized following one AI (Table 7). In successful AIs, insemination volume ranged from 40 to 80 µl using undiluted or diluted semen samples with sperm concentrations from 0.73 to 12.03 million sperm per µl, a sperm motility from 62 to 84% and a sperm viability from 89 to 98% (Table 7).

## Discussion

In this study, semen collection of 20 free-ranging kākāpō on Whenua Hou was successful in 43/46 attempts (93.5%) using both the massage method and a specifically developed bi-polar probe for electric stimulation. This success rate exceeded that of monogamous parrots like macaws (*Anodorhynchus* spp., *Cyanopsitta* spp.; 54.5%), amazon parrots (*Amazona* spp.; 68.7%) or lorikeets (*Lorinae*; 65.5%) [22] and was comparable to that of other non-monogamous psittacines (*Eclectus* spp., *Tanygnathus* spp.) (96.6%) [22]. Success rate of semen collection in birds seems to be strongly seasonal and correlated with breeding behavior [27,43–45]. Here all males were caught during the breeding season in 2019 and were considered as sexually mature (> 5 years), so that semen production was assumed. As wild animals, handling and assisted reproduction could induce stress in kākāpō, potentially resulting in lower success of semen collection. However, previous field studies on Columbian sharp-tailed grouse (*Tympanuchus phasianellus columbianus*) reported a 100% success rate of semen collection [30], suggesting minimal stress-related interference in at least some wild birds.

Electric stimulation was also suitable for semen collection in the kākāpō but showed a tendency (although not statistically significant) for increased rate of sample contamination, particularly with urates, compared to the abdominal massage method. Urates lower semen pH which impairs sperm function. In both mammalian and non-mammalian spermatozoa, various functions such as motility or acrosome reaction are influenced by internal changes of pH [46–48]. In turkeys (*Meleagris gallopavus*), common quail (*Coturnix coturnix*) and chickens (*Gallus gallus f. domestica*), spermatozoa are immotile at low pH-values, while alkaline pH-values increase the percentage and velocity of motile spermatozoa [49]. Previously, the lowest viable pH capable of initiating and supporting motility was determined to be 7.2 for turkey and quail spermatozoa and 7.8 for chicken spermatozoa [49]. In this study, urate-contaminated samples had a lower mean pH (6.9) than uncontaminated ones (7.7), potentially reducing sperm motility after AI, though not statistically confirmed. As sperm motility (MOT and PMOT) is known to be positively correlated with fertilization success [50–52] the occurrence of contaminations was considered to reduce fertilization. Moreover, contaminated semen samples could potentially increase bacterial transmission and may lead to bacterial vaginitis, which is regularly described in poultry [53]. To minimize these risks, only semen samples without macroscopic contamination were used for AI in this study. Considering these results, semen collection in kākāpō should be first tried by massage technique, but when it fails, electric stimulation technique serves as valuable backup, especially in genetically highly valuable males.

Most semen parameters were evaluated in the kākāpō for the first time and may be used as orientation values for further studies. Sperm concentration and viability likely affect fertilization success of AI, especially in polyandrous species, where sperm competition (i.e., competition for fertilization between spermatozoa from different males) is a common phenomenon [54]. In poultry, more than 80% of spermatozoa are ejected from the vagina post mating [55] and only 1% reaches the infundibulum following insertion into the vagina [56]. Furthermore, the outcome of sperm competition is largely determined by sperm concentration and quality [29,57,58], but the mechanism is still not fully understood. In this study, it was assumed that semen from multiple male in the female reproductive tract would enhance competition and fertilization success. Field observations supported this, as egg fertility increased when females copulated multiple times, especially with different males. Consequently, AI was performed using semen from male kākāpō other than the natural mates to increase sperm number and competition. By this means, the number of ejaculates, as well as the total number of spermatozoa in the female reproductive tract was potentially elevated.

Sperm concentration and viability values in polygamous species (e.g., *Eclectus* sp.) ($\overset{―}{x}$ = 3,781,285/µl [c_sp_]; $\overset{―}{x}$ = 94.6% [via]) exceeded those in strictly monogamous species (e.g., macaws) ($\overset{―}{x}$ = 70,068/µl [c_sp_]; $\overset{―}{x}$ = 87.1% [via]). [22]. This is underlined by results of this study on the polygynous kākāpō, where mean sperm concentration ($\overset{―}{x}$ ± SD = 3,279,888.9 ± 3,596,098.6 spermatozoa/µl) and viability ($\overset{―}{x}$ ± SD = 87.4 ± 10.0%) values were slightly lower but comparable to polygamous psittacines. Long sperm also suggests high sperm competition in kākāpō, as spermatozoa have been reported larger in gregarious and sexually dimorphic psittacine species which most likely are facing high levels of sperm competition [59]. Thus, monogamic Southern Festive amazon (*Amazona festiva*) had a lower spermatozoa length (25.45 µm) compared to the polygamous Vasa parrot (*Coracopsis vasa*) (95.43 µm) [59]. Kākāpō, neither being sexually dimorphic nor gregarious, had the fifth longest sperm length (68.33 µm) of more than 60 parrot species examined, indicating a high level of sperm competition [59]. This supports the hypothesis that multiple copulations enhance sperm competition and fertilization probability.

Sperm morphology affects fertilization, as the percentage of viable, morphologically normal spermatozoa (hereafter: MNS) correlates with fertilization success (*r* = 0.66, *p* < 0.01) in the domestic chicken [51]. Morphologic abnormalities are reported to have a negative impact on oocyte penetration (e.g., head abnormalities) [40] and sperm velocity (e.g., tail defects impair movement) [50], while sperm motility (MOT and PMOT) is positively correlated with fertilization success [51,52,58]. However, spermatozoa analyzed in this study exhibited a higher percentage of MNS ($\overset{―}{x}$ ± SD = 43.5 ± 18.3%) as reported in a MSc Thesis in the kākāpō previously ($\overset{―}{x}$ ± SD = 24.1 ± 22.4%; n = 60) [25], but lower compared the other psittacine groups (Amazons: $\overset{―}{x}$ = 51.8%; Cockatoos: $\overset{―}{x}$ = 69.6%; Macaws: $\overset{―}{x}$ = 52.1%; Eclectus parrots: $\overset{―}{x}$ = 70.8%) [19]. The fact that the majority of morphologic alterations were located in the tail ($\overset{―}{x}$ ± SD = 27.1 ± 13.2%) and head ($\overset{―}{x}$ ± SD = 11.0 ± 7.7%) region also corresponds with findings in other studies of psittacines [19]. The lower MNS level in kākāpō contrasts with that of other polyandrous species, where high sperm competition is associated with a high MNS percentage, increasing the likelihood of fathering offspring. This might point towards an impaired semen quality within the Kākāpō, possibly due to low genetic diversity within the kākāpō population [14,25] as previously discussed for the Spix’s macaw [15,60]. This needs to be monitored in further studies to see if in young males resulting from genetically more diverse pairings, the MNS rate is increased.

In a previous study on cockatiels, semen samples stored in modified Lake’s semen extender were stored 120 minutes with higher motility values compared to other semen extenders used [31].This could not be replicated in the kākāpō. Firstly, modified Lake`s diluent resulted in lower mean values of MOT, PMOT and viability compared to other semen extenders. Secondly, MOT, PMOT and viability decreased dramatically during the investigated storage period in Lake`s diluent as well as in most other semen extenders, but was maintained remarkably high with modified Blanco`s semen extender (Table 5). This semen extender had been adjusted in pH and osmolality for cockatiels [31]. However, all mean motility values shortly after semen collection were higher than mean motility results of 34.3% (± 27.8, n = 36) as reported in a previous MSc Thesis in the kākāpō using Lactated Ringers Saline solution as diluent (dilution 1:20) [25]. However, these results should be interpreted with care due to our small sample size and inconsistent time points under field conditions. Nonetheless, they provide a baseline for future kākāpō semen storage research, supporting previous findings that extender effectiveness varies between species, necessitating species-specific adjustments [31].

Artificial insemination was performed 15 times in 12 females in 2019, testing different equipment and techniques. Correct timing of insemination is crucial to achieve optimal fertilization success, ideally performed shortly before or immediately after oviposition [28]. In this study a median volume of 50 µl containing 6,317,500 spermatozoa/µl was used for insemination, assuming that larger volumes with higher TSC could result in higher fertilization rates, according to previous studies in cockatiels [36]. Interestingly, one fertilization was achieved with a comparably low sperm concentration (726.000/µl), but still exceeding the previously reported minimum insemination dose [36]. Natural copulation timing was used to estimate AI timing, though this was imperfect as some females sometimes had 10 days or more between first copulation and egg laying. Insemination post-oviposition seems ideal, however, regularly it is difficult to catch the free-ranging female and to inseminate early enough to fertilize the second egg and concerns about disturbing nesting females limited this approach. To increase the effect of sperm competition, AI after natural copulation seems the best compromise and should be repeated whenever possible.

All females nested and started or continued to lay independent from AI, indicating that capture, handling and AI techniques do not negatively influence nesting behavior. This aligns with findings in both *ex situ* studies in psittacines [20–22] and studies in free-ranging birds [30]. Clutch and egg fertility rates were higher in the AI group compared to the control group. However, a significant difference was not demonstrated in the kākāpō, most likely due to the small sample size - a common issue with threatened species [61]. Egg fertility assessment relied on visual inspection (`candling’), which may underestimate fertility in both groups compared to more advanced egg examination methods such as staining of the perivitelline layer and subsequent fluorescence microscopy [13].

Notably, in the second clutch, egg fertility without AI was 29.41% (5/17), but 70% (14/20) with AI, emphasizing the importance of AI in the kākāpō conservation. A similar outcome was shown in Columbian sharp-tailed grouse, where nest attempt rates increased after the implementation of AR techniques (although not statistically significant) [57]. In both studies, AI success improved over time, likely due to increased familiarity with assisted reproduction techniques and species-specific adaptations. In the present study, five chicks from three different females were verifiably fertilized via AI. This clearly indicates the success of this technique and the potential improvement of the outcome by sperm competition, as a second copulation could be mimicked by AI. Additionally, for the first time ever, a genetically highly valuable male (“Sinbad”, house ID: KM98003), fathered offspring via AI. This emphasises the effectiveness and the importance of assisted reproductive techniques in psittacine species conservation programs as it was already shown previously in the Spix’ Macaw [20] and the St. Vincent amazon [21].

Limitations of the study were the comparably low number of individuals and the low sample size, limiting the number of analyses and the validity of the results. In this regard, the consideration of potential confounders, such as age, environmental factors (e.g., weather conditions—especially rain and sunshine—daylight length, temperature), and nutritional factors (e.g., availability of rimu tree fruits) in the statistical analysis, was not possible due to the small sample size. Furthermore, field conditions limited the number of examinations, explaining why not all samples were analyzed at every time point during semen extender and storage investigations. In general, the selection of males based on genetic importance may have biased results toward a specific subset of the population, limiting broader applicability.

Future studies should investigate semen parameters, semen extenders and storage in a larger sample size to optimize storage conditions and assess their suitability for AI. Cryopreservation of kākāpō semen should be a future goal. Additionally, genetic data should be incorporated to identify factors influencing reproductive success, including gene expression profiling, transcriptomics, and genetic markers affecting semen quality and fertility. Continuous monitoring of potential stress and long-term welfare impacts of frequent handling and assisted reproduction (including electric stimulation) should be implemented.

## Conclusion

Semen collection was successfully performed in free-ranging kākāpō using abdominal massage and electric stimulation techniques and preliminary semen values for orientation and comparison were established for this endangered parrot species. Semen extenders were evaluated and can be used for short-term storage of kākāpō semen without any significant quality losses over a period of 96 hours. Modified Blanco´s semen extender showed the best results. However, the benefit of semen extenders was finally not confirmed, as so far no fertilized eggs were achieved by using diluted semen for artificial insemination (AI). AI in kākāpō resulted in a higher rate of fertile clutches and fertilized eggs, most likely due to the positive effect of sperm competition. Fertile eggs and viable offspring of a genetically high-priority male was achieved directly by artificial insemination, suggesting assisted reproduction as a useful tool in conservation of the endangered avian species such as the kākāpō.
